# Exploring regional and sociodemographic disparities associated with unenrollment for the disease management program for type 2 Diabetes Mellitus using Bayesian spatial modelling

**DOI:** 10.1007/s43999-022-00007-1

**Published:** 2022-08-17

**Authors:** B Kauhl, M Vietzke, J König, M Schönfelder

**Affiliations:** AOK Nordost – Die Gesundheitskasse, Potsdam, Germany

**Keywords:** Disease management program, Type 2 Diabetes Mellitus, Geographic Information Systems (GIS), INLA, Bayesian analysis, AOK Nordost

## Abstract

**Background:**

The disease management program (DMP) for type 2 Diabetes Mellitus (T2DM) is the largest DMP in Germany. Our goal was to analyze regional differences in unenrollment rates, suggest areas for intervention and provide background information, which population groups in which locations are currently not enrolled in the DMP for T2DM.

**Methods:**

In this study, we used data of the 1.7 mil. insurants of the AOK Nordost health insurance. For the visualization of enrollment potential, we used the Besag-York-Mollie model (BYM). The spatial scan statistic (SaTScan) was used to detect areas of unusually high rates of unenrolled diabetics to prioritize areas for intervention. To explore sociodemographic associations, we used Bayesian spatial global regression models. A Spatially varying coefficient model (SVC) revealed in how far the detected associations vary over space.

**Results:**

The proportion of diabetics currently not enrolled in the DMP T2DM was 36.8% in 2019 and varied within northeastern Germany. Local clusters were detected mainly in Mecklenburg-West-Pomerania and Berlin. The main sociodemographic variables associated with unenrollment were female sex, younger age, being unemployed, foreign citizenship, small household size and the proportion of persons commuting to work outside their residential municipality. The SVC model revealed important spatially varying effects for some but not all associations.

**Conclusion:**

Lower socioeconomic status and foreign citizenship had an ubiquitous effect on not being enrolled. The DMP T2DM therefore does currently not reach those population groups, which have a higher risk for secondary diseases and possible avoidable hospitalizations. Logically, future interventions should focus on these groups. Our methodology clearly suggests areas for intervention and points out, which population group in which locations should be specifically approached.

## Background

Disease management programs (DMP) are evidence-based, structured treatment programs to enhance health knowledge and adherence to therapeutic guidelines for patients suffering from chronic diseases [1]. The DMP for type 2 Diabetes Mellitus (T2DM) is the largest DMP in Germany [2]. The idea behind DMPs is to minimize the risk of possible secondary chronic diseases resulting from insufficient treatment for T2DM, such as coronary heart disease, diabetic foot syndrome and glaucoma as well as avoidable hospitalizations and increasing costs [2–4]. In the German healthcare system, there are currently DMPs for Asthma, breast cancer, COPD, type 1 and type 2 Diabetes Mellitus and coronary heart disease [5]. As incentive to encourage physicians and patients to participate within the DMPs, the German health insurance funds receive additional financial compensation if their insurants are enrolled in these DMPs with the aim of improving the quality of care for the chronically ill [6].

While sociodemographic and environmental risk factors for T2DM are widely acknowledged with the elderly and persons with lower socioeconomic status being the most important risk groups [7], little is known about sociodemographic factors associated with diabetics currently unenrolled in the DMPs. Such knowledge is vital, as it would reveal whether the DMPs currently focus on those person groups, which have the largest risk for secondary diseases and avoidable hospitalizations. However, most research in Germany on DMPs focus on the economic effectiveness, quality of care and prevention of secondary diseases [2, 8]. A problem yet to be resolved for all DMPs is the question whether there is selective enrolment in favor of patients with higher socioeconomic status, similar to other voluntary prevention offerings. Only few studies with small number of participants focus on social disparities of DMP enrollment [9]. Although health insurance data provide a rich and detailed data source, this data source has not yet been used to analyze social disparities in DMP enrollment.

Moreover, geographic disparities in enrollment rates have currently not been in the focus, despite strong regional differences [10, 11]. However, visualizing geographic disparities in enrollment rates and providing background knowledge, which population groups in which locations are currently not enrolled is important to establish a framework to better provide information to those population groups, which could benefit most from enrollment into the DMP for T2DM.

Geographic information systems (GIS) and spatial epidemiological methods have been extensively used to identify high risk areas and subsequently identify those population groups, which are most at risk for a wide range of diseases [7, 12, 13]. Additionally, with the increasing availability of geostatistical methods, regression modelling techniques have further developed in recent years to identify not only who is at risk, but more important who is where at risk [7, 13]. The main analytical methods for local regression modelling are Geographically weighted regression (GWR) [7, 13, 14] and Bayesian spatially varying coefficient models (BSVC) [15, 16]. GWR has been previously used mainly for aggregated data e.g. disease rates at the municipality or county level [7, 13], but remains computationally inefficient for analyses of large datasets at the individual level. In contradiction, BSVC models can be used not only for aggregated data, but also for local regression analyses for large-scale individual data [17, 18], providing more detailed insights, which population group is where at risk.

There is evidence that local regression modelling could help to implement more cost effective and practical prevention strategies, when the location-specific risk groups are targeted [7, 13, 19]. In the case of German DMPs however, this modelling strategy could provide information about the location specific sociodemographic factors associated with not being enrolled, resulting in more targeted and cost-effective strategies. The aim of our article is therefore to (i) visualize the proportion of diabetics currently not enrolled in the DMP for T2DM (enrollment potential), (ii) suggest areas for interventions and (iii) provide information, which population group within these intervention areas should be approached for enrollment.

## Methods

### Data

The AOK Nordost covers the three federal states of Berlin, Brandenburg and Mecklenburg-West-Pomerania and is the largest statutory health insurance provider with approximately 25% market share in the region.

For this analysis, we included anonymized data on all individuals that were insured on the first of July, 2019 (1.65 million individuals). We defined insurants with type 2 Diabetes Mellitus as individuals having at least in two quarters of 2019 a laboratory confirmed diagnosis with the ICD-code E11. Of the 1.65 million insurants, 287 thousand (17.3%) had type 2 Diabetes Mellitus and out of those 287 thousand diabetics, 183 thousand (63.8%) were enrolled in the respective disease-management-program.

For the analysis of possible influencing factors, we used at the individual level: Age, sex, being unemployed at 1^st^ July, 2019 as indicator for low socioeconomic status and foreign citizenship. At the aggregated level, we used a commercial dataset of the so-called “Geomarkets” from commercial provider WIGeoGIS. A geomarket consists of approximately 300 households and contains information on demographics, socioeconomic variables, household composition and many more [20]. In total, northeastern Germany consists of approximately 16,500 geomarkets and thus allows a much more detailed analysis than official administrative units such as municipalities or counties. The insurants were aggregated to the geomarkets based on their address coordinates. Several studies have pointed out that area deprivation has an additional adverse ecological effect on individual health outcomes, including DMP enrollment [9, 21]. We therefore calculated a deprivation index based on the variables unemployment rate, proportion of employed persons at the place of residence, purchasing power, persons with high school degree and proportion of persons without formal education. The domains employment, income and education were weighted equally. The resulting index values range from 1 (least deprived) to 100 (most deprived). The methodology is similar to the calculation of the German index of multiple deprivation by Werner Maier [22].

To account for the impact of physician density, we calculated the physician density per 100,000 residents using the gravity model of the LMU Munich, which represents a Germany-specific variant of a 2-step-floating-catchment-area-method (2SFCA). For brevity, we refer for details and the underlying data to previous publications [23].

### Statistical analysis

#### Cartographic visualization of enrollment potential

To be able to visualize the proportion of unenrolled diabetics (enrollment potential) at the level of the 16,500 geomarkets, we used the Besag-York-Mollie (BYM) model. The BYM model is the most commonly used model for displaying rates on small administrative units [24–26]. In its most basic form, the BYM model accounts for varying numbers of insurants per geomarket by weighting the rate within one geomarket to the neighbouring geomarkets and additionally shrinks the rate towards the global mean. Neighbouring areas were defined as areas sharing a common edge or border [24–26]. For the visualization of the enrollment potential, we used the crude number of diabetics not enrolled in the DMP for T2DMP as dependent variable and the total number of expected unenrolled diabetics as offset variable. Since the geomarkets are very small units, especially in urban areas, we further used the stochastic partial differential equation (SPDE) approach to perform an interpolation between the geomarkets to derive a continuous surface of enrollment potential without the problem of artificially created boundaries. The continuous surface was created as we chose not to disclose boundaries of the geomarkets to preserve insurant confidentiality, while still being able to map the enrollment potential at fine geographic scales. This approach has also been used to visualize HIV-prevalence in sub-saharan Africa [27] and reproductive tract infections in Bangladesh at subnational level [28]. The input for this approach consisted of the smoothed rates at the level of geomarkets and their respective centroids. The calculation of the BYM model and the SPDE approach was carried out using the integrated nested Laplace approximation available in the INLA-package for R version 4. We chose the INLA approach over traditional Bayesian inference using Markov chain monte carlo simulations as it does not require long running simulation and sensitivity analyses [29]. The results of the BYM model were then displayed with the R-package ggplot2 [30].

#### Local cluster analysis

Our aim was to pinpoint and suggest specific areas for intervention where the enrollment potential is high. However, a pure prioritization of areas based on the resulting map alone would be error-prone as geomarkets in urban areas are typically very small, whereas geomarkets in rural areas are reasonably large. This would result in important areas with high numbers of currently unenrolled diabetics to be overlooked. To mitigate this issue, the spatial scan statistic (SaTScan) was employed. The spatial scan statistic is a local clustertest, which evaluates the location and the significance of areas where the number of unenrolled diabetics is unusually high. The spatial scan statistic uses a circular scanning window, which is flexible in size and position and evaluates all possible cluster positions and cluster sizes [31]. We restricted the largest possible cluster size to a maximum radius of 30 km [7]. This was done to detect reasonably sized clusters as the population density especially in Mecklenburg-West-Pomerania is very low and the standard setting would produce results of no practical use. We used a purely spatial Poisson model with a maximum radius of 30 km. Statistical significance was calculated based on 9,999 Monte-Carlo replications. The calculation was carried out using the SaTScan software and the results were then imported in R for visual representation.

### Regression modelling

#### Global regression modelling

In this study, we used two different classes of logistic regression models: Global and local regression models. First, we used a global non-spatial regression model using the following explanatory variables at the individual insurant level: Sex and age, being unemployed at the date of 1^st^ of July, 2019 and foreign citizenship. At the level of geomarkets, we used our deprivation index, average household size, percentage of persons commuting to work outside their residential municipality and physicians per 100,000 residents.

We started with a non-spatial global regression model to check for multicollinearity using the HH-package in R. The HH-package assigns a variance inflation factor (VIF) to all explanatory variables within the regression model. A VIF > 5 indicates the presence of multicollinearity and warrants the removal of one or more of the explanatory variables [32].

Since the data for this study were collected at very fine geographic scales, we further evaluated if the inclusion of spatial relationships provided further improvement of the parameter estimates. For the global model, we evaluated three different priors: (i) unstructured spatial effects where it is assumed that no spatial autocorrelation exists and possible interactions between the administrative units are random and follow no spatial structure. (ii) The second model includes a spatially structured effect, following the assumption that close objects are more similar than distant objects (spatial autocorrelation). In the third model (iii), we accounted for spatially structured and random effects and thus a combination of the former two effects. This was performed in the INLA-package in R, by assigning an iid prior for model (i), a Besag prior for model (ii) and a Besag-York-Mollie (BYM) prior for model (iii) [24]. The neighbourhood matrix was the same that was used for the disease mapping model and is based on the 16,500 geomarkets of northeastern Germany. The deviance information criterion (DIC) was then computed to allow a comparison between the three global spatial regression models. The DIC and AIC can then be compared to find the best fitting model.

#### Local regression model

The global regression models calculate only one single regression coefficient per explanatory variable and therefore assume that the strength of association is equal across the study area. However, our study area consists of the capital city of Berlin and thus includes Germany`s largest city, which in itself differs drastically between its neighborhoods. Contrary to Berlin, Brandenburg and Mecklenburg-West-Pomerania are the federal states with some of the lowest population densities in Germany and differ drastically in their demographic and socioeconomic composition from Berlin. A global regression model for these three states thus could possibly reflect only a coarse estimation but will not necessarily reflect the true underlying process within a subregion of our study area. As shown in previous studies, local regression models, such as Geogaphically weighted regression (GWR) provide a more detailed insight into the relationships between various diseases and their explanatory variables [7, 33]. While GWR is typically used for aggregated, ecological analyses or analyses based on sample points of environmental variables [7, 33, 34], it is currently not suitable for analyses of large datasets with several hundreds of thousand observations. We therefore used a Bayesian spatially varying coefficient model (BSVC) within the INLA package, which is capable of handling large datasets computationally efficient while allowing the computation of regression coefficients for each administrative unit [17, 18]. The input consisted of the same variables as in the global regression models, where the information from the geomarkets was assigned to the individual insurant based on their respective address coordinate. To make the analysis more computationally efficient, we used a neighborhood matrix based on the municipalities of northeastern Germany and the neighborhoods of the larger cities Berlin, Potsdam, Frankfurt (Oder), Schwerin, Rostock and Cottbus instead of the neighborhood matrix of the geomarkets. Calculating the spatially varying coefficients based on 1,700 administrative units instead of 16,500 drastically reduced computing time from several days to several hours. Additionally, the continuous variables (age, deprivation, commuters) were standardized to allow a direct comparison, which variable in which region is of relative importance. To allow an estimation how reliable the local coefficients are, we additional calculated the probability that the coefficients are > 1 (exceedance probability), except for the variable household size, where we calculated the probability that the coefficients are < 1, as the local model indicated a negative and ubiquitous relationship between unenrollment and household size [17].

## Results

### Spatial distribution of DMP enrollment potential

**Fig. 1:**
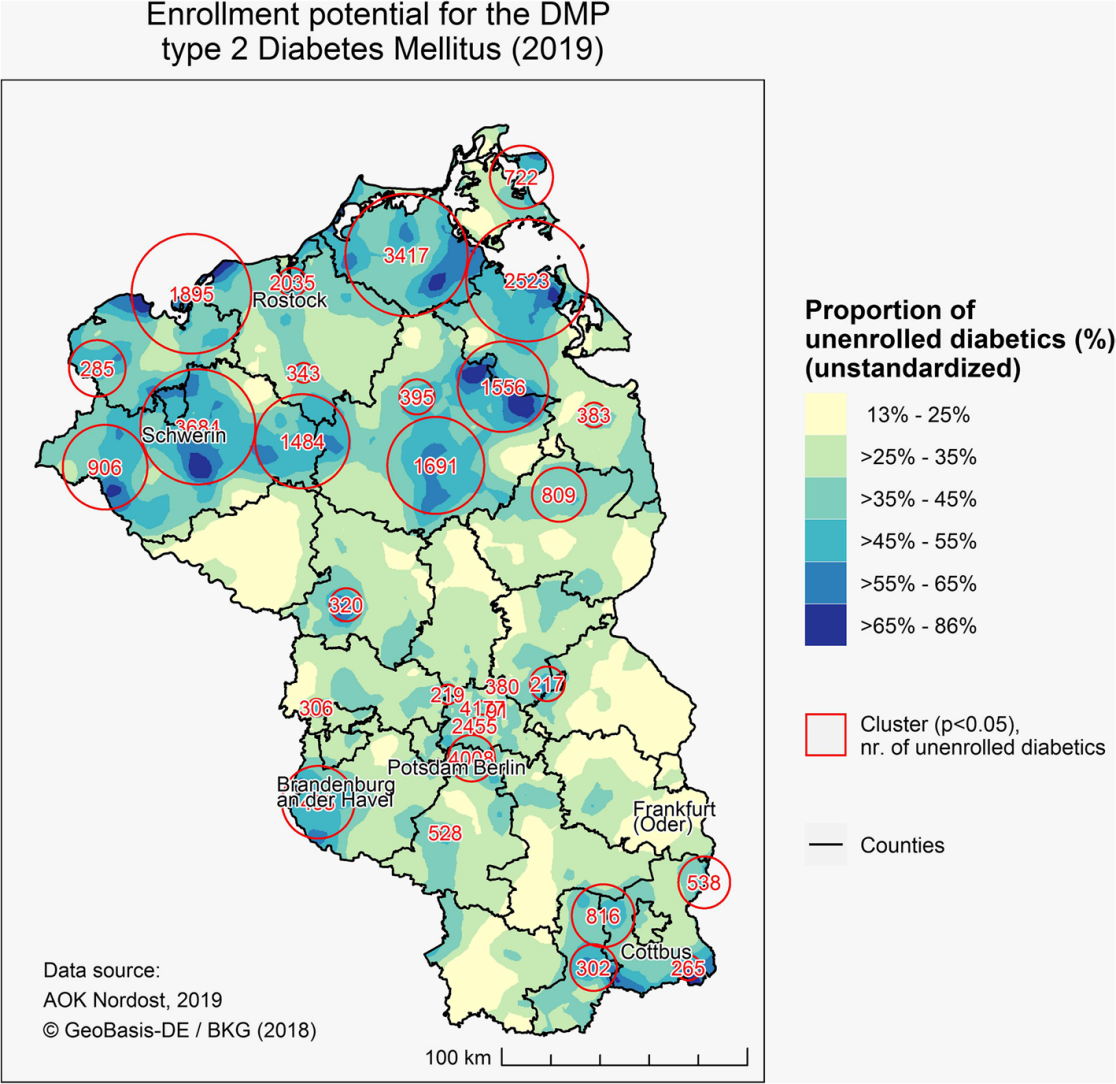
Enrollment potential for the DMP for type 2 Diabetes Mellitus

The global average of DMP-enrollment for T2DM was 63.2% and logically the global average of enrollment potential 36.8% in 2019. The rates with the highest enrollment potential and significant clusters with high numbers of unenrolled diabetics were mainly found in Mecklenburg-West-Pomerania, although smaller clusters were also detected in Brandenburg and Berlin (Fig. 1). It is important to note that the clusters with the largest number of unenrolled diabetics were found in Berlin and the neighboring Teltow, south of Berlin.

### Global regression model

The model with structured and unstructured effects had the best model fit (Table 1). According to the VIF, no multiollinearity was detected between the explanatory variables.

**Table 1:** Goodness-of-fit statistics for the evaluated regression models

Model	Prior	DIC
Unstructured effect	Iid	359,575
Structured effects	Besag	357,210
Unstructured and structured effects	Besag-York-Mollie (BYM)	357,189
Spatially varying coefficients	Besag	359,995

Male persons had a 6.2% lower risk of not being enrolled than women (Table 2). With every year of age, the risk of not being enrolled decreased by 0.3%. Insurants with foreign citizenship had a 5.8% higher chance not to be enrolled than insurants with German citizenship. Currently unemployed insurants had a 10% higher risk not to be enrolled than insurants with other types of insurance. Area deprivation had no significant impact on enrollment status. With an increase in household size decreased the risk of not being enrolled: With one person more per household, the risk of not being enrolled decreased by 19.9%. An increase of 1% of commuters decreased the risk of not being enrolled by 0.4%. The physician density had no significant effect on enrollment status for the DMP T2DM.

**Table 2:** Regression coefficients for unenrolled diabetics

Variable	Coefficient	CI 2.5%	CI 97.5%
Intercept	1,327	1,077	1,637
Sex: male (ref: female)	0,938	0,923	0,953
Age	0,997	0,996	0,998
Foreign citizenship (ref: German cit.)	1,058	1,022	1,094
Unemployed (ref: not unemployed)	1,100	1,063	1,139
Deprivation	1,000	0,999	1,001
Household size	0,801	0,770	0,834
Commuters (%)	0,996	0,994	0,998
Physician density (per 100,000)	0,998	0,996	1,001

### Local regression model

The local regression model had a higher DIC than the global regression models.

**Fig. 2:**
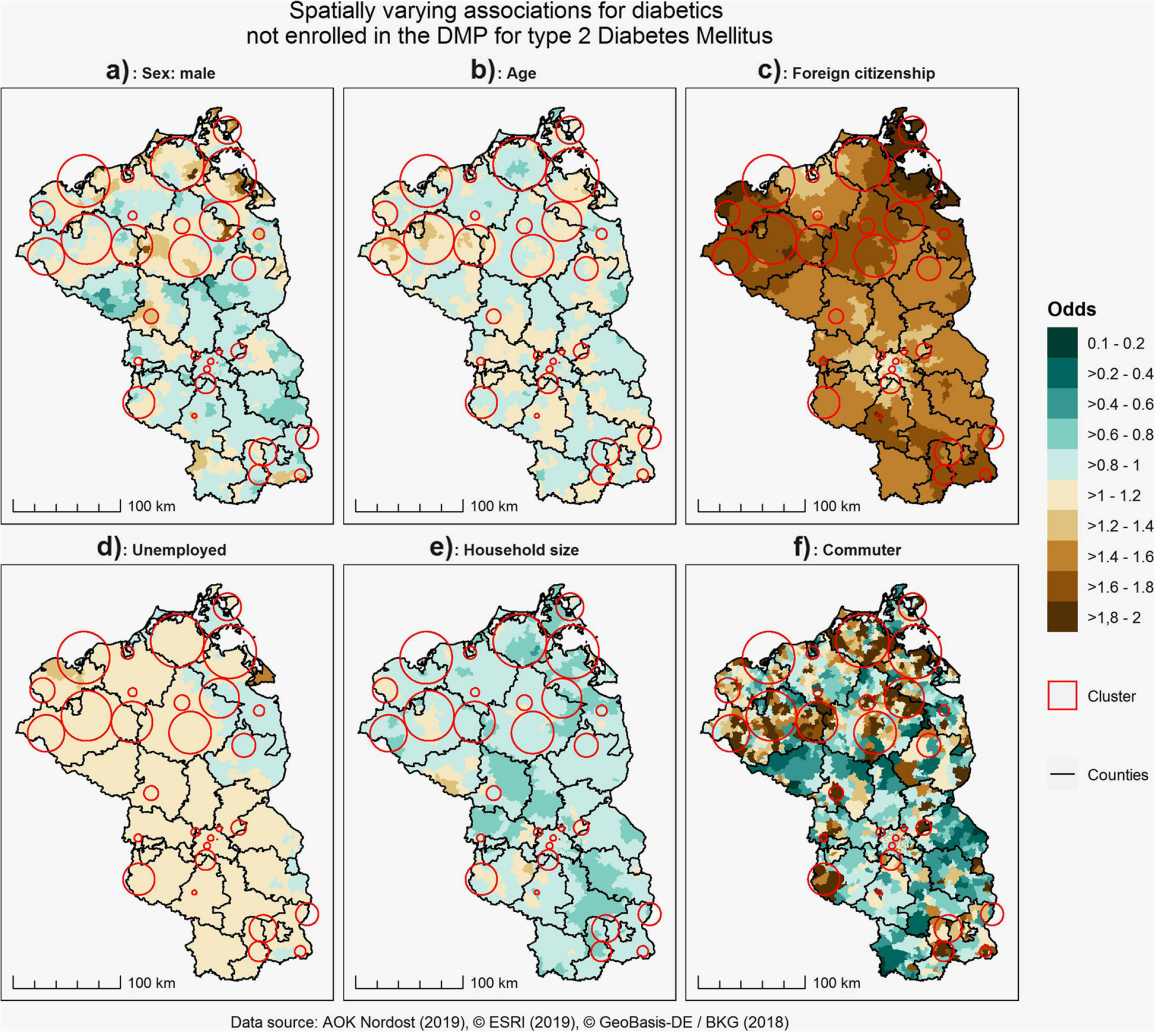
Spatially varying coefficients for currently unenrolled diabetics in the DMP for type 2 Diabetes Mellitus

**Fig. 3:**
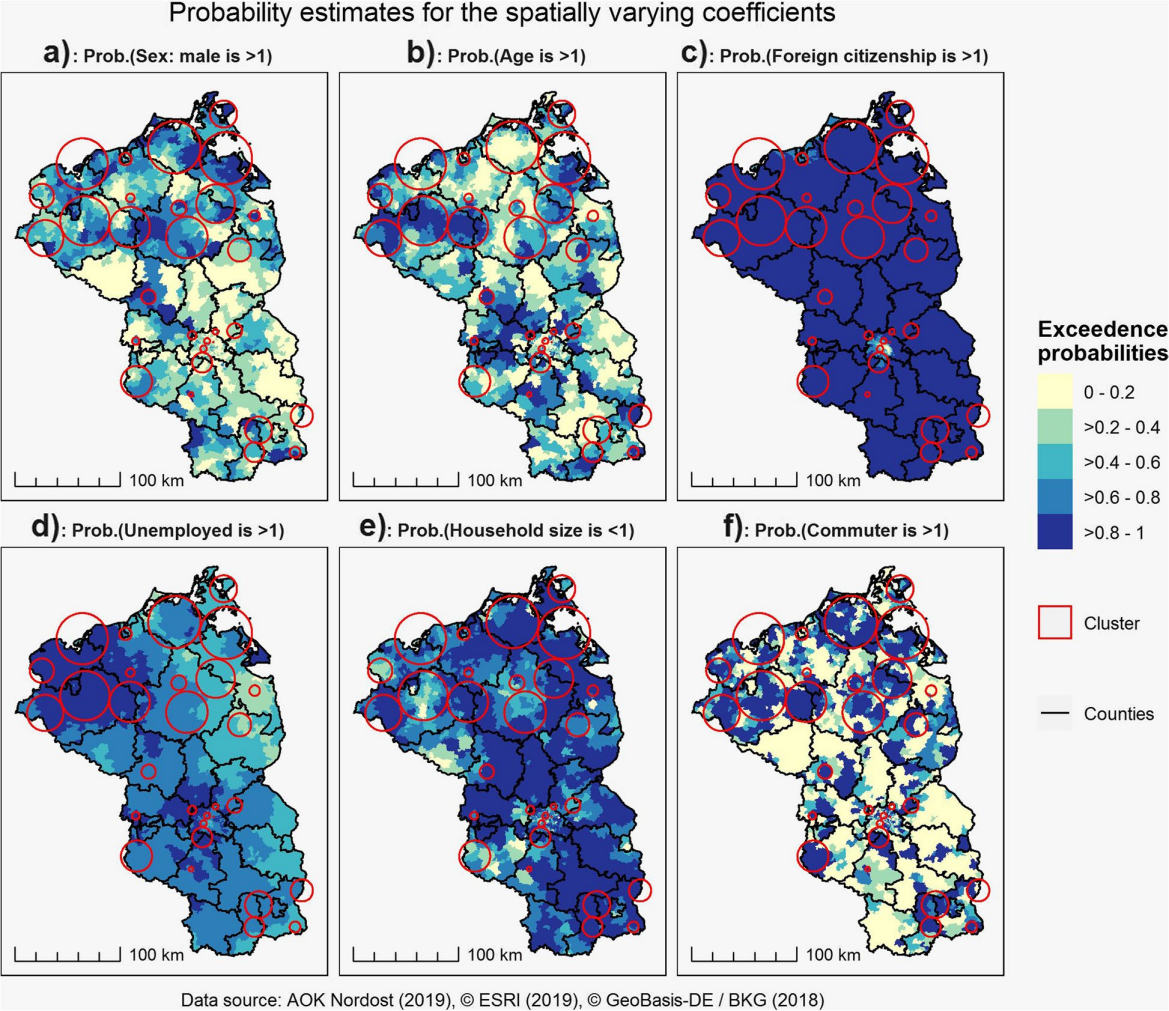
Probability maps for the spatially varying coefficients

For several variables, we could observe important regional differences in the strength of association (Fig. 2). The probability maps for the local coefficients are shown in Fig. 3. In the majority of clusters in Mecklenburg-West-Pomerania, males had a higher chance not to be enrolled than women with a probability of more than 60 to 80%. In contradiction, in the clusters in Berlin and Teltow, men had a lower chance not be enrolled than women. Similarly, an increase in age had a positive effect on not being enrolled in the majority of clusters in Mecklenburg-West-Pomerania, but also in a small fraction of neighborhoods in Berlin and Teltow. The probability that age had a positive effect in these clusters was more than 80%. Only in a small fraction of clusters in Brandenburg, age had a negative effect on not being enrolled. Foreign citizenship had – except for the center of Berlin – overall a positive effect on not being enrolled with a probability of more than 80%, with the strongest effect in predominantly rural areas in Mecklenburg-West-Pomerania and parts of Brandenburg. It is important to note however, that these areas generally have a small proportion of inhabitants with foreign citizenship and thus the effect is strong, but affects only a small proportion of insurants. Being currently unemployed had a comprehensive and positive effect on not being enrolled except for several municipalities close to the Polish-German border. The probability that being unemployed had a positive effect was with more than 80% highest in the northwestern part of Mecklenburg-West-Pomerania and the surrounding municipalities of Berlin. The effect of household size was – with the exception of a small fraction of municipalities – negatively associated with not being enrolled. The highest probabilities for a negative association with more than 80% were highest in the majority of municipalities in Brandenburg and Mecklenburg-West-Pomerania. Strong regional differences could be observed in the association between persons commuting to work outside their respective residential municipalities. However, a positive association could be observed in the majority of clusters. In these clusters, the probability for a positive association was 80% and higher.

## Discussion

This is likely one of the first studies analyzing social disparities of T2DM DMP enrollment using geostatistical methods based on large-scale health insurance data.

We found strong regional differences and local clusters for enrollment potential for the DMP T2DM. Similarly, we found that especially unemployment, foreign citizenship and household size had an almost ubiquitous effect on enrollment potential, while sex, age and the proportion of inhabitants commuting to work had a regionally varying effect on enrollment potential. The deprivation of the respective place of residence did not have a significant effect on unenrollment for the DMP T2DM and neither did the physician density.

The overall enrollment rate was 63.2%, which is lower than a previous study based on data of the association of statutory physicians Northrhine (Kassenärztliche Vereinigung Nordhrein) [10], but higher than estimated based on a study in Bavaria [11]. This is not surprising, as we could observe marked geographic differences in enrollment potential within our study area, ranging from 13 to 86% (corresponding to enrollment rates of 14% to 87%), respectively.

Our results are partly in line with other studies, revealing that individual lower socioeconomic status was associated with unenrollment. This partly reflects results of the BARMER health insurance, where it was found that participants of the DMP T2DM had a slightly larger proportion of persons with higher educational levels as compared to non-participants [6].

While our study is in line with the aforementioned results of the BARMER, our results partly contradict previous research on DMP enrollment for coronary heart disease (CHD): A previous study revealed no significant socioeconomic disparities on DMP enrollment for CHD at the individual level and only little influence of deprivation of the place of residence [9]. In contradiction, our results clearly revealed that individual unemployment had a considerable influence on T2DM DMP unenrollment, while we could not observe a significant association to deprivation of the place of residence. Logically, individual level socioeconomic status tends to be more important than deprivation of the place of residence in our study. With regard to sex, our results are in line with previous research demonstrating that males are more likely to participate in the DMP CHD [35].

Based on existing research, there is currently little information available on the role of household size, commuters and persons with migration background. However, when compared to existing research on socioeconomic factors influencing participation in prevention campaigns, our results show comparable tendencies. Based on research of the Robert Koch-Institute, persons with lower socioeconomic status are less likely to participate. Likewise, persons living together with a partner are more likely to participate than persons living alone. This corresponds to our findings that unemployed persons are more likely not to be enrolled in the DMP T2DM. Additionally, persons living in smaller households are more likely not to be enrolled, which corresponds to findings of the RKI that persons living together with a partner are more likely to participate in prevention campaigns [36]. While certainly DMP enrollment is not comparable to participation in prevention strategies, the tendencies are clear: Persons with lower socioeconomic status are less likely to participate in preventive offerings, which potentially enhance their individual health status.

Several studies point out that there is selective enrollment in DMPs [6, 9]. There is a strong debate, whether DMPs actually benefit the participants, with some studies confirming a positive effect, while other studies find no significant benefit [8]. Based on our findings that persons with lower socioeconomic status are more likely not to be enrolled, we can confirm that selective enrollment exists. As a result, the DMP T2DM does not reach those persons groups, that have a considerable higher risk for secondary diseases and potentially avoidable hospitalizations, but rather reaches those person groups, which would have a higher health literacy and higher participation in prevention campaigns anyway [6, 9, 36]. Given these findings, future evaluations about the effectiveness of DMPs should adjust for socioeconomic status of participants, as this is likely to have a major influence on adherence to therapeutic guidelines and logically on the effectiveness of DMPs in general.

Based on our results, it is possible to suggest following acquisition guidelines: Sex and age depends on the respective location. In some areas, men and in some areas women should be addressed. While certainly all unenrolled diabetics should be informed about the benefits of DMP enrollment, the effect of age on enrollment potential differs by location. This might be a valuable background information for GPs in the area where to focus more on diabetics under or over 72 years of age as the mean age of diabetics in our study is 72 years. Except for the center of Berlin, persons with foreign citizenship should be targeted, especially in predominantly rural areas. Except for areas close to the German-Polish border, unemployed persons are also a focus group. Since household size was negatively associated, especially persons living alone may be considered as additional target groups. For persons commuting to work outside their residential municipality, the identified clusters are important: It is advisable to target especially commuters in the identified spatial clusters as the scaled coefficients show a relative importance of commuters in relation to the other sociodemographic factors.

### Limitations

Since our study is based on a geostatistical analysis of health insurance routine data, we have to acknowledge several limitations associated with health insurance data and the chosen geostatistical methods:

Although the AOK Nordost is northeastern Germany`s largest statutory health insurance provider, our results are not necessarily representative for the whole population. Large sociodemographic differences exist between the health insurance providers with the AOK Nordost having a bigger proportion of older persons and persons with a lower socioeconomic status [37]. As a result, the effect of deprivation is likely to be underestimated as the proportion of AOK Nordost insurants is higher in deprived areas.

Since we selected all persons insured at the 1^st^ of July, 2019, we did not evaluate how long the respective insurant has a diagnosis of T2DM or is enrolled in the DMP. As our focus was not in the evaluation of the effectiveness of the DMP, but rather on sociodemographic factors associated with (un)enrollment, this limitation will most likely not influence the results drastically, since especially chronically ill persons have longer insurance times with the AOK Nordost than persons not suffering from chronic diseases.

The variables household size and percentage of commuters were taken from aggregated data of the whole population. It is possible that the detected associations do not necessarily reflect associations on an individual insurant-level. However, since we analyzed at microgeographic scales, it is likely that this problem is rather small as the geomarkets represent approximately 300 households on average. Therefore, the ecological bias is most likely rather small as compared to the normally used counties, most spatial epidemiological research in Germany focuses on [38–40].

Another limitation is that we could only focus on individual insurant and area-level characteristics but could not include information on the attitude towards DMPs of the general physicians in the area. It is possible that there are differences in the attitudes of the physicians regarding the DMP T2DM, influencing the enrollment rates in the respective regions [41].

Several studies find a positive association between DMP enrollment and various markers for disease progression, health literacy and economic effectiveness, while other studies are indecisive of the positive effect of DMPs [1, 2, 8]. This debate was not the focus of our study. We would like to point out however, given the sociodemographic disparities of (un)enrollment, that future studies should strongly include several measures of socioeconomic status to adjust for selective enrollment to really be able to evaluate the effectiveness of the DMPs.

From a methodological perspective, the application of a BSVC provided more detailed insights than traditional global regression models, despite the fact that the DIC for the local model was higher than for the global regression models. Nonetheless, we think that the information who is where currently not enrolled is more useful than only the information, who is not enrolled, even when the goodness-of-fit-statistics suggest a better performance for a global model [18]. The application of a local model therefore provided more detailed insights than the standard global regression models. However, we do have to note for some variables, e.g. sex, age, foreign citizenship and being currently unemployed, that the coefficients show only moderate regional differences, while for the variable proportion of persons commuting to work, the coefficients show strong regional differences. This could be seen as a strength of the BSVC over the traditional GWR, which generally tends to display the same amount of regional variation for all included variables in the regression model. However, regardless of the chosen methodology for local regression modelling, it is currently not feasible to capture the true regional variability of the strength of association and the resulting local coefficients remain estimates. However, given the strong sociodemographic differences within our study area, spatially varying differences in the association between enrollment potential and sociodemographic variables are more realistic than only one single coefficient per explanatory variable as estimated by global regression models [7, 15].

## Conclusion

This is likely the most detailed geostatistical analysis of enrollment potential for the DMP T2DM. We found strong regional differences in enrollment potential. Similarly, our findings confirm that persons with lower socioeconomic status and foreign citizenship are more likely not to be enrolled. The DMP T2DM therefore does currently not reach those population groups, which have a higher risk for secondary diseases and avoidable hospitalizations. Based on the chosen geostatistical methods, we provided information, which population groups in which locations should be targeted to increase enrollment for the DMP T2DM. These results could result in more targeted and therefore cost effective strategies to directly approach those population groups, which could benefit most from structured treatment for T2DM.

## Data Availability

The data used in this study contains sensitive information of a health insurance provider (social data). Social data are part of social secrecy (§ 35 SGB I) and have to be kept secret by federal law (§ 78 SGB X). The data may therefore not be made available to third parties.

## Abbreviations

DMP: Disease management program
T2DM: Type 2 diabetes mellitus
BYM: Besag-York-Mollie model
GWR: Geographically weighted regression
BSVC: Bayesian spatially varying coefficient model
CHD: Coronary heart disease
GIS: Geographic information systems
INLA: Integrated nested laplace approximation
ICD: International classification of diseases
2SFCA: 2-Step-floating-catchment-area-method
SPDE: Stochastic partial differential equation
VIF: Variance inflation factor
AIC: Akaike`s information criterion
DIC: Deviance information criterion
